# When the Ostrich-Algorithm Fails: Blanking Method Affects Spike Train Statistics

**DOI:** 10.3389/fnins.2018.00293

**Published:** 2018-04-30

**Authors:** Kevin Joseph, Soheil Mottaghi, Olaf Christ, Thomas J. Feuerstein, Ulrich G. Hofmann

**Affiliations:** ^1^Section for Neuroelectronic Systems, Clinic for Neurosurgery, Medical Center- University of Freiburg, Faculty of Medicine, University of Freiburg, Freiburg, Germany; ^2^Freiburg Institute for Advanced Studies, University of Freiburg, Freiburg, Germany

**Keywords:** stimulation, blanking circuit, stimulation artifact, electrophysiology, spike detection

## Abstract

Modern electroceuticals are bound to employ the usage of electrical high frequency (130–180 Hz) stimulation carried out under closed loop control, most prominent in the case of movement disorders. However, particular challenges are faced when electrical recordings of neuronal tissue are carried out during high frequency electrical stimulation, both *in-vivo* and *in-vitro*. This stimulation produces undesired artifacts and can render the recorded signal only partially useful. The extent of these artifacts is often reduced by temporarily grounding the recording input during stimulation pulses. In the following study, we quantify the effects of this method, “blanking,” on the spike count and spike train statistics. Starting from a theoretical standpoint, we calculate a loss in the absolute number of action potentials, depending on: width of the blanking window, frequency of stimulation, and intrinsic neuronal activity. These calculations were then corroborated by actual high signal to noise ratio (SNR) single cell recordings. We state that, for clinically relevant frequencies of 130 Hz (used for movement disorders) and realistic blanking windows of 2 ms, up to 27% of actual existing spikes are lost. We strongly advice cautioned use of the blanking method when spike rate quantification is attempted.

**Impact statement:**

Blanking (artifact removal by temporarily grounding input), depending on recording parameters, can lead to significant spike loss. Very careful use of blanking circuits is advised.

## Introduction

Electrical stimulation of neuronal tissue is used to provide insights into neuronal responses (Penfield, 1958; Brindley and Lewin, 1968; McCreery et al., 1990; Tehovnik, 1996), to influence brain networks (Pezaris and Reid, 2007; Xie et al., 2014; Choi et al., 2016), and to alleviate clinically relevant symptoms of neurological disorders (Kandel et al., 2000; Tronnier et al., 2002). To evoke a response, an electrical charge is delivered via electrodes to the region of interest, as described in seminal work by Rattay (1989) and Tehovnik et al. (2006). Deep Brain Stimulation (DBS) with high frequencies (HFS), which has similar effects to lesioning (Benabid et al., 1991), but is not irreversible, has gained popularity since the 90's. It is now considered to be an effective, symptomatic treatment for diseases such as Parkinson's or Obsessive Compulsive Disorder (OCD) (Nuttin et al., 1999; Aouizerate et al., 2004; Abelson et al., 2005). HFS is understood to work by inhibiting neuronal activity in the stimulated brain region (McIntyre et al., 2004) or due to extracellular release of GABA (Hiller et al., 2007; Xie et al., 2014). HFS has also been shown to affect other neurotransmitter release (Lee et al., 2004; Deniau et al., 2010; Joseph et al., 2015).

Unfortunately, while synchronously attempting the “read-out” of neuron's electrophysiological responses during HFS, the obligatory high level of amplification leads to signal contamination or even frequent saturation of recording systems, i.e., the stimulation artifact (Hashimoto et al., 2002). The overlapping artifact may render the acquired neuronal signal difficult to analyze, which can be debilitating when neuronal spike statistics or responses are the focus of the investigation (Wagenaar and Potter, 2002).

Stimulation artifact removal techniques within recorded data has been a thriving field, where linear and non-linear filtering (Sennels et al., 1997; Parsa et al., 1998; Gnadt et al., 2003; Whittington et al., 2003, 2005) or artifact template subtraction utilizing a number of methods (Zhiyue and McCallum, 1998; Hashimoto et al., 2002; ter Braack et al., 2013) have been employed with mixed results.

Alternatively, several manufacturers of recording systems (Plexon Inc.—USA, Multi-Channel Systems—Germany) have implemented a hardware solution to prevent amplifier saturation by temporarily grounding inputs using a “blanking circuit.” The input stage of the amplifier is thus protected from saturation and even damage, with a trigger signal that marks the stimulation pulse. The amplifiers are reconnected to the circuit after a user defined a “blanking window,” based on manufacturer recommendations. It is evident that during the “blanking window,” the signal values are set to zero, thus masking valuable neuronal signals.

This protective blanking window needs to have a temporal onset at least at the start of the actual stimulus pulse and needs to cover the complete discharge period of the surrogate circuit of both the probe and electronics, which helps avoid cross talk between the stimulation and recording electrodes. This “artifact free” signal is then further analyzed for changes in LFP or spike content (Heffer and Fallon, 2008; ter Braack et al., 2013; Yi et al., 2013; Multichannel Systems, 2015; Qiu et al., 2015).

However, we caution users to exercise extreme care when using this seemingly bullet-proof method, as the chosen settings may very well have an influence on spike quantifications and indiscriminate use may introduce significant errors in real world spike count recordings for basic neuroscience (Georgopoulos et al., 1986) and neuroengineering (Serruya et al., 2002; Schwartz, 2004; Hochberg et al., 2006).

There exists a probability that the events of interest overlap with the blanking window, which could result in event loss and detrimental consequences for subsequent neurobiological conclusions, requiring additional means to validate experiments (Xie et al., 2014). The following study investigates the influence of this widely used technique from both a theoretical and experimental standpoint.

## Methods

### Theoretical evaluation—probability of data loss

**Figure 1:**
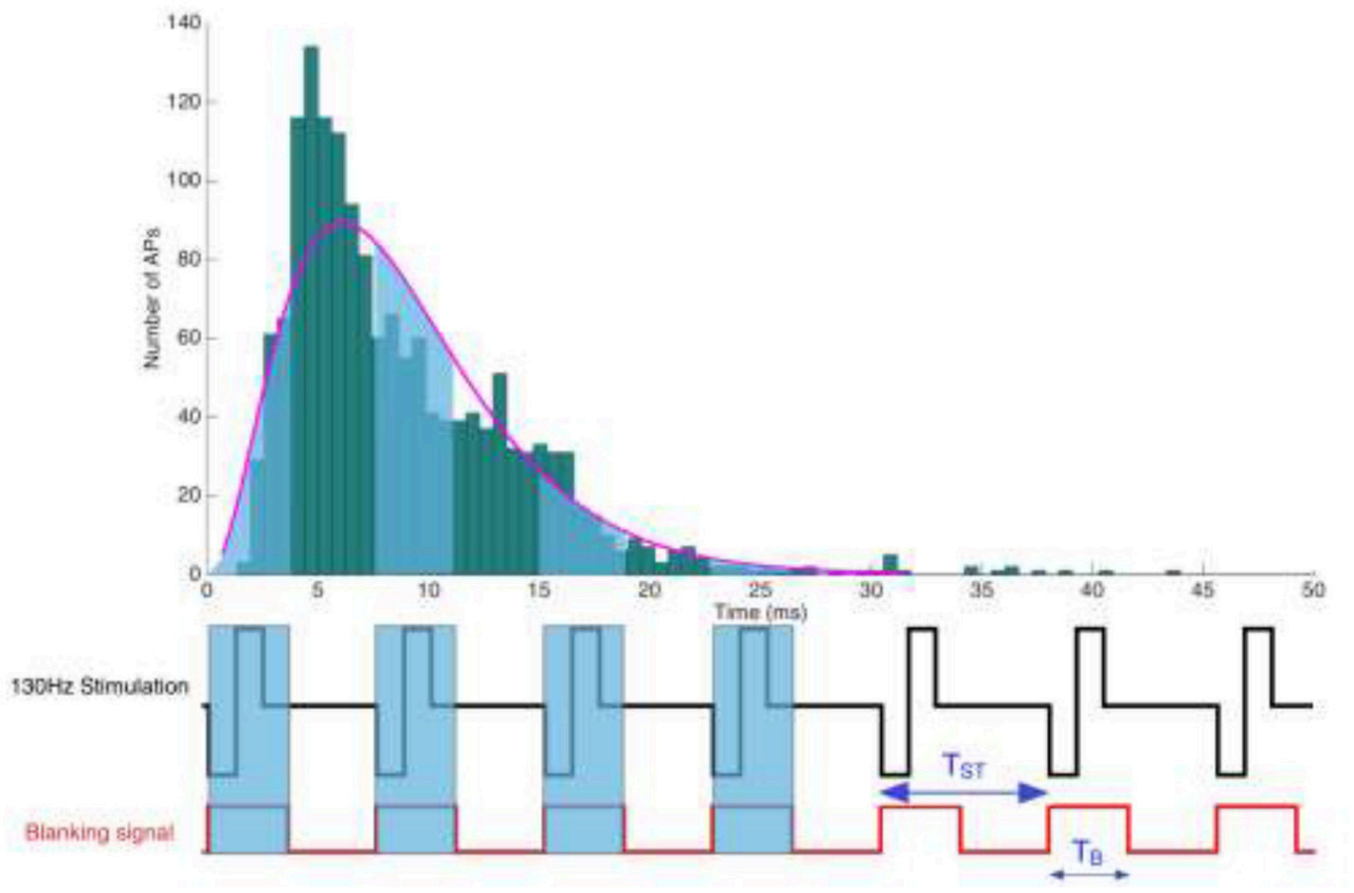
Illustration of a periodic rectangular stimulation pattern with a repetition period *T*_*ST*_. (Below) Representation of a stimulation pulse train and the corresponding blanking signal, depicting its temporal extension beyond the actual stimulation pulse. Blanking windows are meant to suppress electrical signals thought to endanger sensitive amplifiers.

To assess the potential impact of blanking on electrophysiological results based on spike statistics and counts, we utilize the spike probability of cells under investigation based on their interspike-interval-distribution. As previously described, spiking activity of neurons can be by a Poisson distribution and the interspike-interval-distribution (ISI) can be modeled by a Gamma distribution (Bair et al., 1994) (Figure 1). Cellular properties then define the ISI and thus variability is to be expected between consecutive spikes.

The stimulation pulse triggered blanking circuit is designed to not just mask the actual pulse artifact with a width of *PW* and a repetition frequency *F*_ST_, but needs to include an additional safety margin to outlast the capacitive discharge, which could originate from either the system design or the electrode-tissue coupling. Setting the stimulation pulse width *PW*, a safety margin *T*_M_ and the stimulation's repetition frequency *F*_ST_, we seek to estimate the likelihood of a spike to fall within the blanking window *T*_B_ = (*PW*+*T*_M_), where it will be lost.

The likelihood of a spike falling within TB is determined by the actual spiking probability of the neuron type under investigation, which is modeled by the Gamma distribution (Equation 1), defined with scale and shape parameters, a and b, respectively. The Gamma function Γ (a) and the mean value of the distribution μ are defined using Equations (2, 3).

(1) $$\begin{array}{rcl} f\left(x,a,b\right) & = & \frac{1}{\Gamma \left(a\right)b}{\left(\frac{x}{b}\right)}^{a-1}e^{\frac{-x}{b}} \end{array}$$

(2) $$\begin{array}{rcl} \Gamma \left(k\right) & = & \int_{0}^{\infty }x^{k-1}e^{-x}dx \end{array}$$

(3) $$\begin{array}{rcl} \mu & = & a.b \end{array}$$

**Figure 2:**
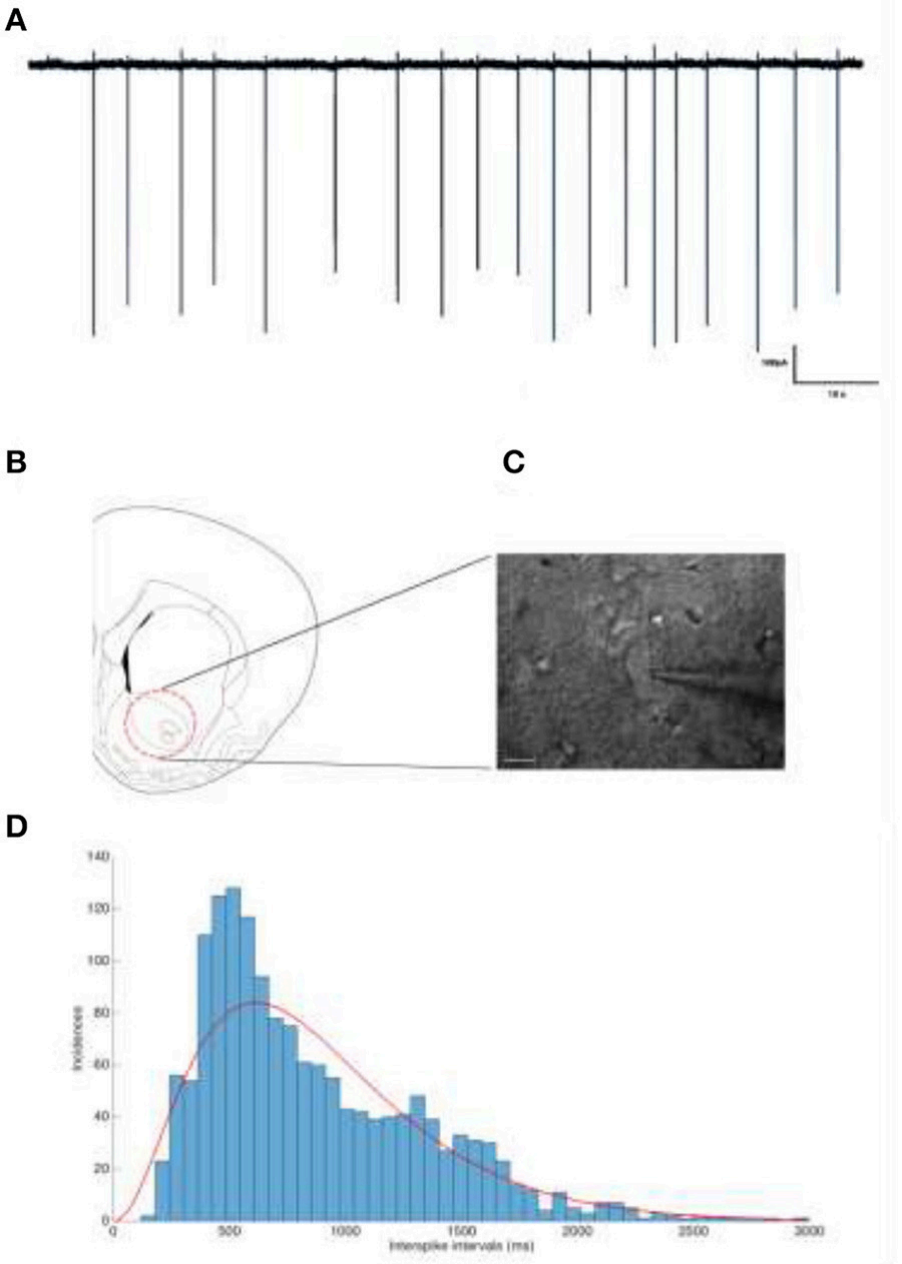
**(A)** Representative trace of a tonically firing cholinergic interneuron. The spiking frequency is ~1.12 Hz. **(B)** The neurons of interested were located and recorded from the nucleus accumbens. **(C)** IR-DIC image of a patched cholinergic interneuron. Scale bar is 20 μm. **(D)** Interspike interval distribution of Cholinergic interneurons show a Gamma distribution.

The cumulative probability of the Gamma distribution between x1 and x2 can be calculated using Equations (4, 5). The maximal number of blanked bins *N*_*b*_ is determined using Equation 6, which integrates the ISI distribution of up to 99.95% of the counted events. If the number of blanked regions multiplied by the blanking window is larger than this x99.95 count, no spikes will be detected, as in Equation (7). The maximum probability of detection of non-blanked events is given by Equations (8, 9). Figure 2D illustrates blanking windows within the fitted Gamma distribution.

(4) $$\begin{array}{r} P\left(\Delta x,a,b\right)=\int_{x1}^{x2}f\left(t,a,b\right)dt \end{array}$$

(5) $$\begin{array}{r} P\left(x_{99.95},a,b\right)=\int_{0}^{x_{99.95}}f\left(t,a,b\right)dt=0.9995 \end{array}$$

(6) $$\begin{array}{r} N_{b}=\left\lfloor \frac{x_{99.95}}{T_{ST}}\right\rfloor \end{array}$$

Then

(7) $$\begin{array}{r} N_{b}*T_{B}>x_{99.95} \end{array}$$

(8) $$\begin{array}{r} P_{Detectable_{APs}}=0 \end{array}$$

(9) $$P_{Detectable_{APs}}\leq \;\sum_{n=1}^{N_{BW}}\int_{x_{n}}^{x_{n}+T_{B}}f(t,a,b)dt$$

### Experimental validation—actual data loss

To validate our theoretical findings, we performed cell-attached patch clamp recordings of cholinergic interneurons from the nucleus accumbens of rats in a slice preparation. This provides high SNR recordings with action potentials easily identified, despite non-saturating stimulus artifacts (Figure 2B) providing ground truth measurements for spike detection and counts. Under these conditions, the stimulation artifact is clearly visible without obscuring the events, establishing a well-controlled test bed for the quantification of the blanking artifact. Inspite of the usage of rectangular pulses for the HFS, the non-rectangular stimulation artifact observed is as a result of effect of aliasing. Aliasing occurs when the sampling frequency is not high enough for the perfect digitization and reconstruction of the artifact. Since a rectangular pulse has infinite frequencies, it is close to impossible to increase the sampling frequency high enough to reconstruct the stimulation pulses.

#### Animals and tissue preparation

All protocols were approved by the responsible Animal Care Committee of the Regierungspräsidium Freiburg (Permit X16/02A), and all efforts were made to minimize the number of animals used, with respect to statistical constraints. Male Wistar rats (3–4 weeks old) (Janvier, France), housed in groups of 4, under standard lighting (12 h light-dark cycle), 22°C and 40% humidity were used in this study, and were allowed access to food and water *ad libitum*.

The rats were decapitated after an overdose of inhalation anesthetic (Forene, Baxter, USA), and the brains were quickly extracted and submerged in oxygenated ice-cold sucrose aCSF (artificial cerebrospinal fluid). Slices (300 μm) were prepared using a vibratome (VT 1200, Leica, Germany) containing the nucleus accumbens with coordinates previously described (Varatharajan et al., 2015). After incubation at 37°C for 30 min in sucrose fortified aCSF, and a resting phase of 20 min at room temperature, the slices were ready for patch clamp experiments. All experiments were carried out at 32°C, controlled by an inline heater (Multi Channel Systems, Reutlingen, Germany) (Stuart et al., 1993). Neurons were visualized at 40 × using infrared differential interference contrast (IR-DIC) video microscopy (XM-10, Olympus Corporation, Germany) (Figure 2B). Tissue slices were perfused with oxygenated aCSF containing: (in mM) 125 NaCl, 25 NaHCO3, 2.5 KCl, 1.25 NaH2PO4, 1 MgCl2, 2 CaCl2, and 25 Glucose (pH 7.4 with 5% CO2).

#### Electrophysiological recordings

Recordings were carried out using a Multiclamp 700B Amplifier (Molecular Devices, USA) and the data was digitized using a CED 1401 Mark II (Cambridge Electronic devices, UK), acquired using a custom routine in IGOR Pro (Wavemetrics Inc., USA). Cholinergic interneurons from the Nucleus accumbens were chosen for their tonic firing behavior (Xie et al., 2014) (Figures 2B,C). The distribution of inter-spike-intevals of the cholinergic cells used in this study is shown in Figure 2D which can be modeled by a Gamma distribution (red line) in accordance with (Dayan and Abbott, 2001).

Slices were submerged in the recording chamber and continuously perfused (4 ml/min) with oxygenated aCSF (34°C). Recording electrodes were pulled from thick walled borosilicate glass capillaries (2.0 mm O.D. and 1.2 mm I.D), filled with 150 mM NaCl (3–5 MΩ) (Perkins, 2006). To prevent clogging of the electrode tip while approaching targeted cells, a positive pressure was applied (40 mbar). Once a dimple was observed on the cell membrane surface, the pressure was released and a GΩ seal was seen to form almost instantly. If not, light suction pulses were applied until the seal was obtained. To prevent artificial depolarization of the cell membrane, the voltage was clamped such that a 0 μA holding current was maintained, as described previously (Perkins, 2006). Action potentials could be observed almost instantaneously, a feature to be expected from tonically firing cholinergic interneurons (Figure 2A).

#### Electrical stimulation

Bipolar platinum-iridium (Pt/Ir) electrodes (GBCBG30, FHC Inc., Maine) with a central cathode (Diameter: 75 μm, Area: 0.0062 mm^2^) and a concentric anode were used for stimulation at a distance of 500 μm from the cell soma. The stimulation was carried out either using a commercially available PlexStim (Plexon Inc., USA) or a stimulator developed in-house (Mottaghi and Hofmann, 2015; Mottaghi et al., 2015) used to generate biphasic electrical stimuli with amplitude of 12 μA.

**Figure 3:**
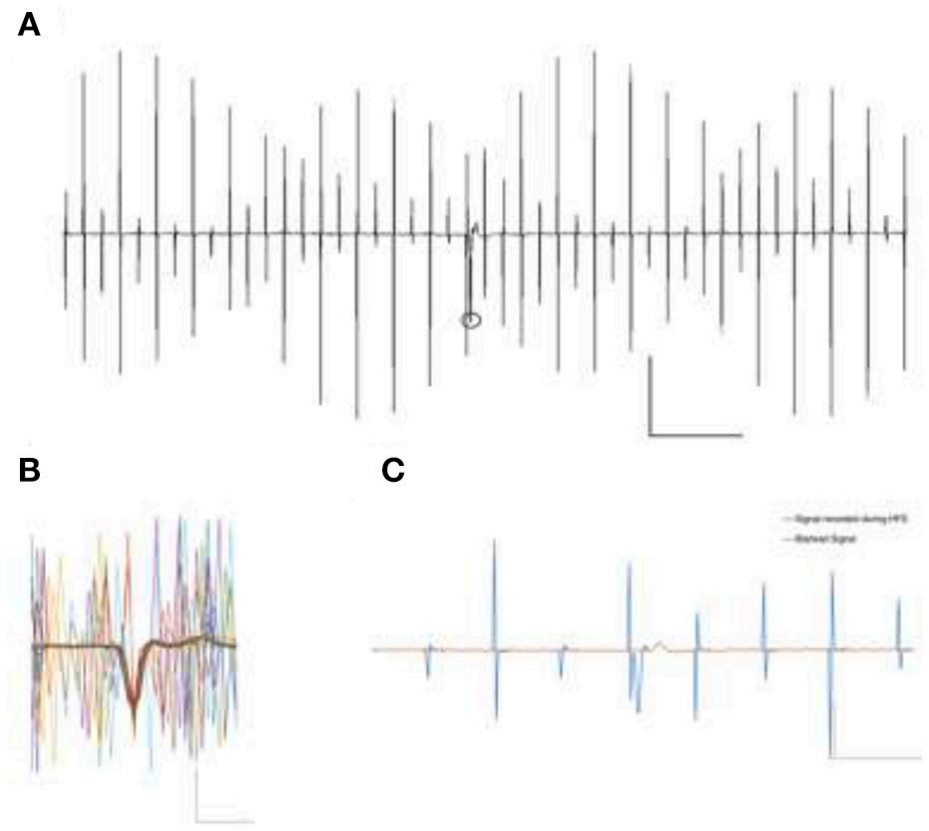
**(A)** Signal contamination during the application of 130 Hz electrical rectangular waveform stimulation. Stimulation parameters were biphasic, 130 Hz, 12 μA, 65 μs pulse window. The circle represents a detected event in spite of contaminating artifact. Scale X: 500 pA, Y:20 μs. **(B)** Overlay of some possible occurrences of stimulation artifacts, in themselves not able to veil the prominent spike Example trace of intrinsic firing spikes under the depicted cell-attached patch clamp recording. Scale X: 500 pA, Y:1 μs. **(C)** Stimulation contaminated cell attached spike recording. A blanking window of 1.5 ms very effectively removes all the stimulation artifacts but obviously shrouds parts of the spike as well (red line). Automated spike detection was then ineffective to detect the clipped spike in the blanked signal. Scale X: 500 pA, Y: 5 μs.

Stimulation parameters were chosen based on therapeutic High Frequency Stimulation (HFS) as described previously (Varatharajan et al., 2015). Both stimulators were used in charge-balanced mode and the pulse width was controlled on-line with an oscilloscope (TPS2012, Tektronix, Switzerland) in parallel to the electrode. Stimulation runs of 100 s each were performed with a constant stimulation current of 12 μA, at a distance of 500 μm. Stimulation produces a visible artifact in the cell-attached extracellular recordings, despite the charge balanced stimulation (Figure 3A).

#### Experimental paradigm

Experimental runs were divided into phases of 100 s each. Spike detection and counting was carried out offline in Matlab (Mathworks, USA) by an algorithm taking spike shapes into account. The algorithm is based on the detection of three main features of recorded events i.e., amplitude, rise-fall slopes, and the peak shape, defined during baseline recording. Based on arbitrarily chosen, overlapping signal windows, the algorithm detects potential events of interest despite artifact contamination by using the previously defined parameters. It then counts and archives the detected events for further statistical analysis. After each detection step, the event count was quantified and confirmed by a blinded user to be compared against the detected events. Figure 3B illustrates a detected event during the high frequency stimulation phase and an overlay of 67 distinct event contaminations to visualize possible artifact positions relative to the spikes. After baseline recordings (100 s), the cells were stimulated for 100 s and then allowed to rest for 300 s before further experiments were carried out. Each cell was measured in triplicate.

## Results

The cell attached recording technique was chosen to obtain the best signal-to-noise ratio for action potentials despite ongoing electrical stimulation, yet did not protect against contamination. Even charge-balanced stimulation pulses frequently overlap action potentials with substantial amplitude (Figures 3A,B).

Signal deterioration, however, is worsened by setting all signal values to zero for the duration of the blanking window *T*_*B*_, as can be seen with an exemplary spike in Figure 3C. It illustrates the successful removal of the stimulation artifacts at the cost of a clear change in event parameters due to the inconsiderate use of the blanking mechanism. Such distorted events are generally not detected by baseline-oriented methods like the one utilized here.

**Figure 4:**
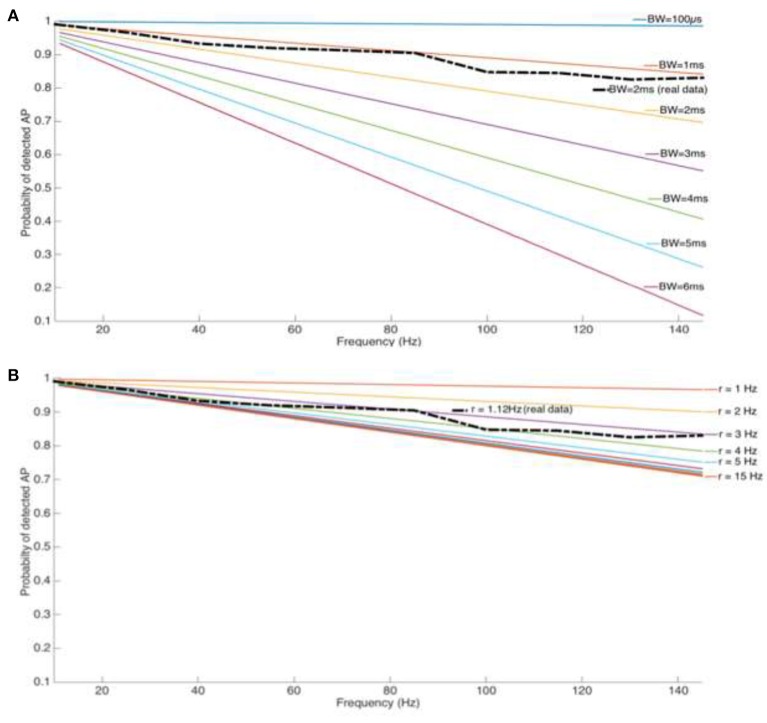
**(A)** Loss of spike count for a tonic firing cell type (Cholinergic interneuron from the Nucleus Accumbens) and stimulation pulse width P_w_ but increasing blanking window length and stimulation frequency. **(B)** Loss of spike count for a reasonable blanking window (T_b_ = 2 ms) and increasing stimulation frequency and spike rate.

**Figure 5:**
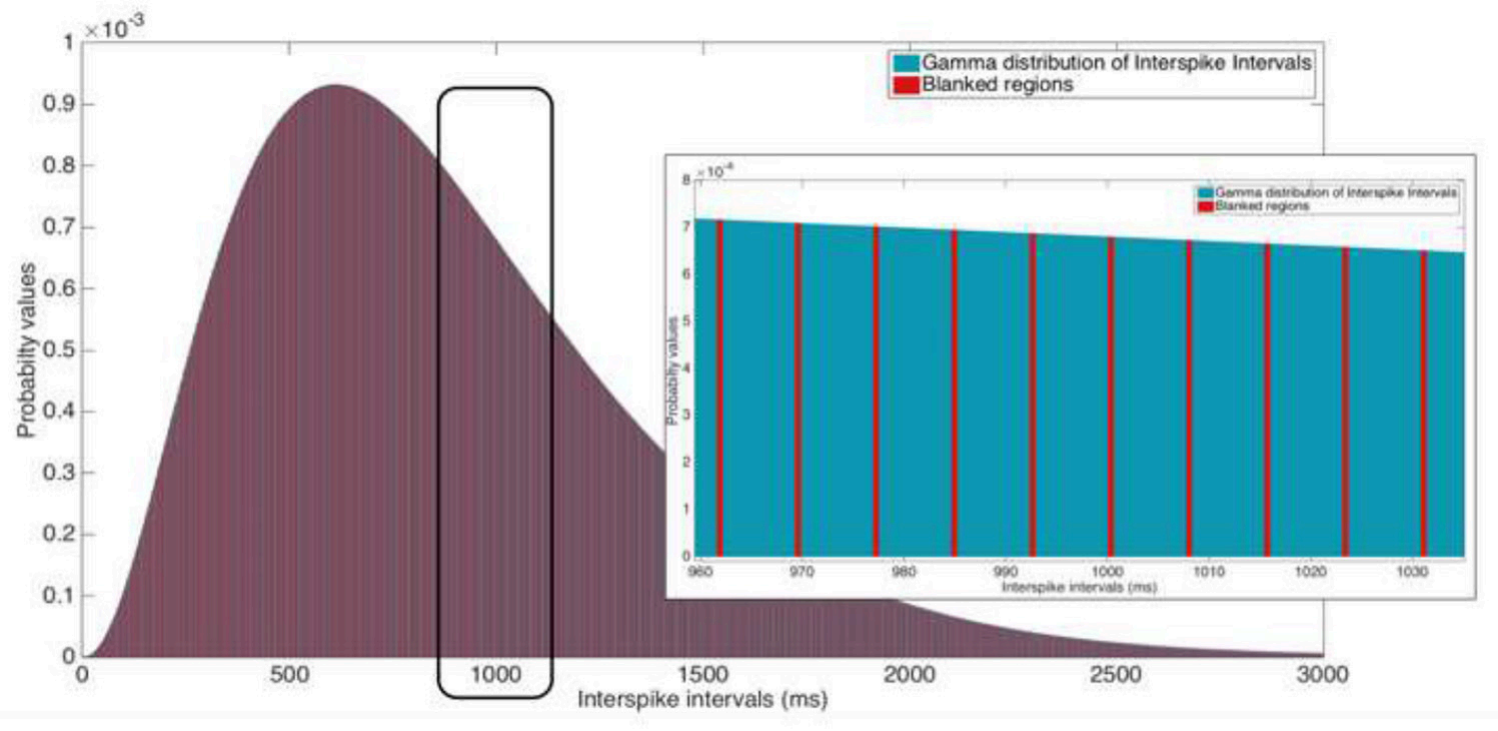
Illustration of a 2 ms blanking window suppressing 130 Hz stimulation. Inset: Zoomed view of the ISI that ranges from 960 to 1,035 ms. Ordinate scales are equal for both the ISI distribution and the inset image.

As the above theoretical considerations illustrate, the usage of a blanking window will result in event loss, as given by Equation (9) (Figure 5). The event loss for different blanking window widths is dependent on the stimulation/blanking frequency (Figures 4A,B). The most commonly used blanking window of 2 ms leads to a spike loss rate of up to 30 %, at therapeutically relevant frequencies. Wider blanking windows, motivated by sensitive amplifiers with longer saturation periods, clearly aggravates the situation and event loss may reach up to 80%.

Any potential loss in event counts is influenced by electrophysiological properties of the cells under investigation, given by their firing rates. Although intuitive, the percentage of lost events is directly proportional to the spike rate. However, for cell types with a spike rate >3 Hz, the loss is independent of the spike rate and is influenced by the stimulation frequency alone. The length of the blanking window and the intrinsic mean of cells' spiking rate are two parameters that play an important role in detectable spikes while recording during electrical stimulation with blanking system on.

Figure 4A depicts a comparison of the blanking window over a range of 200 μs−6 ms, while Figure 4B illustrates a scenario with spiking rates from 1 to 15 Hz. A real life situation is overlaid on both figures with an average spiking rate of *r* = 1.12 Hz found from the cholinergic cells in this study, and blanking windows of 2 ms as a commonly used parameter (dashed-line).

## Discussion and conclusion

When it comes to utilizing electrical stimulation in the nervous system, electrophysiological recordings from an increasing number of neurons and an increasing number of experimental settings are in danger of being corrupted by the infamous stimulation artifact. The currently used gold standard of blanking the recording system during stimulation pulses to make the amplifiers invulnerable against saturation and potential damage may very well-introduce unexpected errors in precision analysis and reconstruction. Starting from theoretical considerations, we urge to a very cautious and well-reflected use of blanking as it inadvertently introduces loss of information by shrouding perfectly well-defined spikes relevant for statistics.

Even though it seems evident that the usage of any type of blanking circuit in real spike recordings is to be with great caution and its effects need to be studied prior to use, this report is, to our knowledge, the first one to quantify the detrimental effect blanking may have with respect to the actual event counts at clinically relevant stimulation frequencies. We approached this quantification by the known theoretical description of firing activity based on neuronal interspike-interval histograms. Blanking nullifies the very histogram bins, which coincide with the blanking window period and width within this distribution. As the histogram can be modeled by a Gamma function, it is possible to estimate the number of spikes affected, depending on the window width *T*_*B*_, its frequency *F*_*ST*_ and the average firing rate R, of the cell type under consideration. As the Gamma function is not limited in time, we restrict calculations to the 99.95 percentile and have to report on loosing up to 27% of spikes with a quite common blanking window of 2 ms and therapeutically relevant 130 Hz stimulation.

In order to validate our theoretical evaluation, we used high SNR data from cell attached recordings of cholinergic interneurons from the nucleus accumbens of rats. They were collected in context of a critical re-assessment of the effects of HFS in slices (Xie et al., 2014) and corroborate the theoretical results of substantial spike loss by blanking within the error margins of our automated spike detector.

In laboratory settings, where the mechanism of action is being explored, alternative stimulation modalities such as optogenetic stimulation can be used, even at high frequencies, to examine the effects on firing rates (Deisseroth, 2015; Huidobro et al., 2017, 2018). However, even this modality comes with its own difficulties and traps (Ayling et al., 2009; Cardin et al., 2009; Han et al., 2009).

In conclusion, we urge establishing confidence in spike statistics obtained during electrical stimulation by taking independent safeguards like pharmacological controls and careful experimental design. This is a critical and delicate issue which entails that all aspects of the experiment, analysis and conclusion in a study are accounted for based on the techniques and hardware used. Blanking, for example in this study, is a procedure, designed by the device manufacturer as a safety tool, which has been demonstrated to influence the outcome of a whole experiment potentially leading to false conclusions if not taken into consideration.

## Author contributions

SM and KJ conducted experimental and theoretical work; TF and UH supervised the experimental and theoretical work; OC provided technical support; SM, KJ, OC, TF, and UH wrote and reviewed the manuscript.

### Conflict of interest statement

The authors declare that the research was conducted in the absence of any commercial or financial relationships that could be construed as a potential conflict of interest.
